# A small-scale fractionation pipeline for rapid analysis of seed mucilage characteristics

**DOI:** 10.1186/s13007-020-00569-6

**Published:** 2020-02-24

**Authors:** James M. Cowley, Lina Herliana, Kylie A. Neumann, Silvano Ciani, Virna Cerne, Rachel A. Burton

**Affiliations:** 1 Australian Research Council Centre of Excellence in Plant Cell Walls, School of Agriculture, Food and Wine, University of Adelaide, Waite Campus, Urrbrae, SA Australia; 2 Australian Research Council Centre of Excellence in Plant Energy Biology, School of Agriculture, Food and Wine, University of Adelaide, Waite Campus, Urrbrae, SA Australia; 3 Dr. Schär R&D Centre, AREA Science Park, Padriciano 99, 34149 Trieste, Italy

**Keywords:** Mucilage, Myxospermy, Extraction, Polysaccharide, *Plantago ovata*, Flax, Chia, Psyllium

## Abstract

**Background:**

Myxospermy is a process by which the external surfaces of seeds of many plant species produce mucilage—a polysaccharide-rich gel with numerous fundamental research and industrial applications. Due to its functional properties the mucilage can be difficult to remove from the seed and established methods for mucilage extraction are often incomplete, time-consuming and unnecessarily wasteful of precious seed stocks.

**Results:**

Here we tested the efficacy of several established protocols for seed mucilage extraction and then downsized and adapted the most effective elements into a rapid, small-scale extraction and analysis pipeline. Within 4 h, three chemically- and functionally-distinct mucilage fractions were obtained from myxospermous seeds. These fractions were used to study natural variation and demonstrate structure–function links, to screen for known mucilage quality markers in a field trial, and to identify research and industry-relevant lines from a large mutant population.

**Conclusion:**

The use of this pipeline allows rapid analysis of mucilage characteristics from diverse myxospermous germplasm which can contribute to fundamental research into mucilage production and properties, quality testing for industrial manufacturing, and progressing breeding efforts in myxospermous crops.

## Background

In a process called myxospermy, seeds of many plants produce viscous polysaccharide gels called mucilage when imbibed in water. The mucilage of *Arabidopsis thaliana* has often been used as a proxy for studying cell wall biosynthesis [1–8]. More recently other myxospermous species like *Linum usitatissimum* and *Plantago ovata* have also been adopted as genetic models [9–14] revealing the utility that novel systems can have in unravelling complex synthetic pathways. Furthermore, these novel model systems have the added benefit of being directly commercially-relevant. *P. ovata* (psyllium) and *L. usitatissimum* (flaxseed) mucilage are used as gums with varied applications in the food and health industries. Both are used as natural food structuring ingredients and gluten replacements [15–18] and are rich sources of dietary fibre shown to prevent various gastrointestinal diseases [19–21]. A comprehensive myxospermous model system would allow gene-structure–function links to be made but there remains a technical disconnect between these facets. The functional study of myxospermous species preceded their use as genetic models and the scale and precision of the extraction techniques have generally not been updated since. A significant number of researchers use the methods of Sharma and Koul [22], Balke and Diosady [23], or similar. These methods are simple, effective and robust, using a magnetic stirrer to heat and agitate a seed/water mixture followed by straining to isolate released mucilage from seeds. However, there are several technical issues that limit the use of these techniques in screening applications. Firstly, the techniques are not high-throughput, generally requiring 3–4 h to process a single sample (per magnetic stirrer). Secondly, the quantity of mucilage produced is excessive for downstream chromatographic and yield analyses which require milligram-scale quantities or less. Thirdly, the techniques often offer incomplete extraction, leaving a significant amount of mucilage adhered to the seed. It is also important to note that seed mucilage is not homogenous. Its multi-layered nature is evident simply by visual inspection of stained expanded mucilage in nearly all species [24] The dual-layered nature of *Arabidopsis thaliana* mucilage has been the basis of many studies on cell wall polysaccharide biosynthesis [25] and Yu et al. [26–28] have recently highlighted the importance of fractionating mucilage to effectively unravel structural differences that underlie polysaccharide functionality.

Here we describe a pipeline suitable for the rapid extraction and fractionation of quantities of seed mucilage suitable for yield and chromatographic analyses. Within four hours, three chemically- and functionally-distinct fractions can be isolated from 24 samples per shaking incubator and the use of a shaking incubator allows adjustment in time, temperature and fractionation profile. The utility of this pipeline is demonstrated through its ability to: identify intergeneric and interspecific variation in seed mucilage extractability, yield and composition, screen for known quality parameters in field-grown myxospermous samples, and identify lines of interest from germplasm collections.

## Methods

### Materials

*Arabidopsis thaliana* seeds (ecotype *Columbia-0*) were grown as per Tucker et al. [29]. Flax (*Linum usitatissimum*) and chia (*Salvia hispanica*) seeds were purchased from Woolworths (Frewville, South Australia). *Plantago ovata* and *Plantago cunninghamii* seeds were obtained and bulked from sources listed in Phan et al. [30]. *P*. *ovata* varieties were grown in field trials conducted in 2017 and 2018 in Kununurra, Western Australia. Gamma-irradiated *P. ovata* mutants used for germplasm screening were obtained from a glasshouse-grown population described previously [10].

Once harvested or purchased, all seeds were dried at 37 °C for at least 72 h and then stored in sealed containers at room temperature until analysis.

### Reagents and solutions

Ruthenium red hydrate (#C075) was purchased from ProSciTech, Australia and the staining solution was prepared at 0.01% w/v in water following Arsovski et al. [5]. To prevent bubble formation on the seed surfaces during imaging, the staining solution was sonicated for 5 min under vacuum to remove dissolved gases. KOH and HCl (Sigma-Aldrich) were made to a 0.2 M solution in water.

### Mucilage staining

Expanded seed mucilage was observed in situ following Arsovski et al. [5] to compare and validate extraction techniques. After positioning seeds on a microscope slide (Rowe GM2715, Australia) staining solution was added beneath a coverglass (ProSciTech No. 1, Australia) and images were captured on a dissecting microscope (Zeiss Stemi 2000-C, Germany) equipped with a colour digital camera (Zeiss AxioCam ERc 5s, Germany).

### Conventional seed mucilage extraction techniques

The effectiveness of total mucilage extraction by the fractionation pipeline described here was validated against previously published methods by Balke and Diosady [23], Yu et al. [26], Sharma and Koul [22], and Voiniciuc et al. [4]. Balke and Diosady’s method (also used previously by our group, Phan et al. [30]) is simple, stirring seeds and water heated to 80 °C on a magnetic stirrer for 90 min after which the liberated mucilage is strained through nylon mesh to remove seeds. Yu et al*.*’s method uses an extended extraction (4 h) with RT 0.2 M solution of KOH. Sharma and Koul’s method combines seeds with dilute acid (0.2 M HCl) in a conical flask, which is stirred on a heated magnetic stirrer until the seeds have changed colour (20 min). Seeds are strained through nylon mesh and washed twice with hot water. Finally, Voiniciuc’s method uses the physical force (30 Hz for 30 min) of a tissue disruptor-type mixer mill.

### Rapid small-scale mucilage fractionation pipeline

**Fig. 1:**
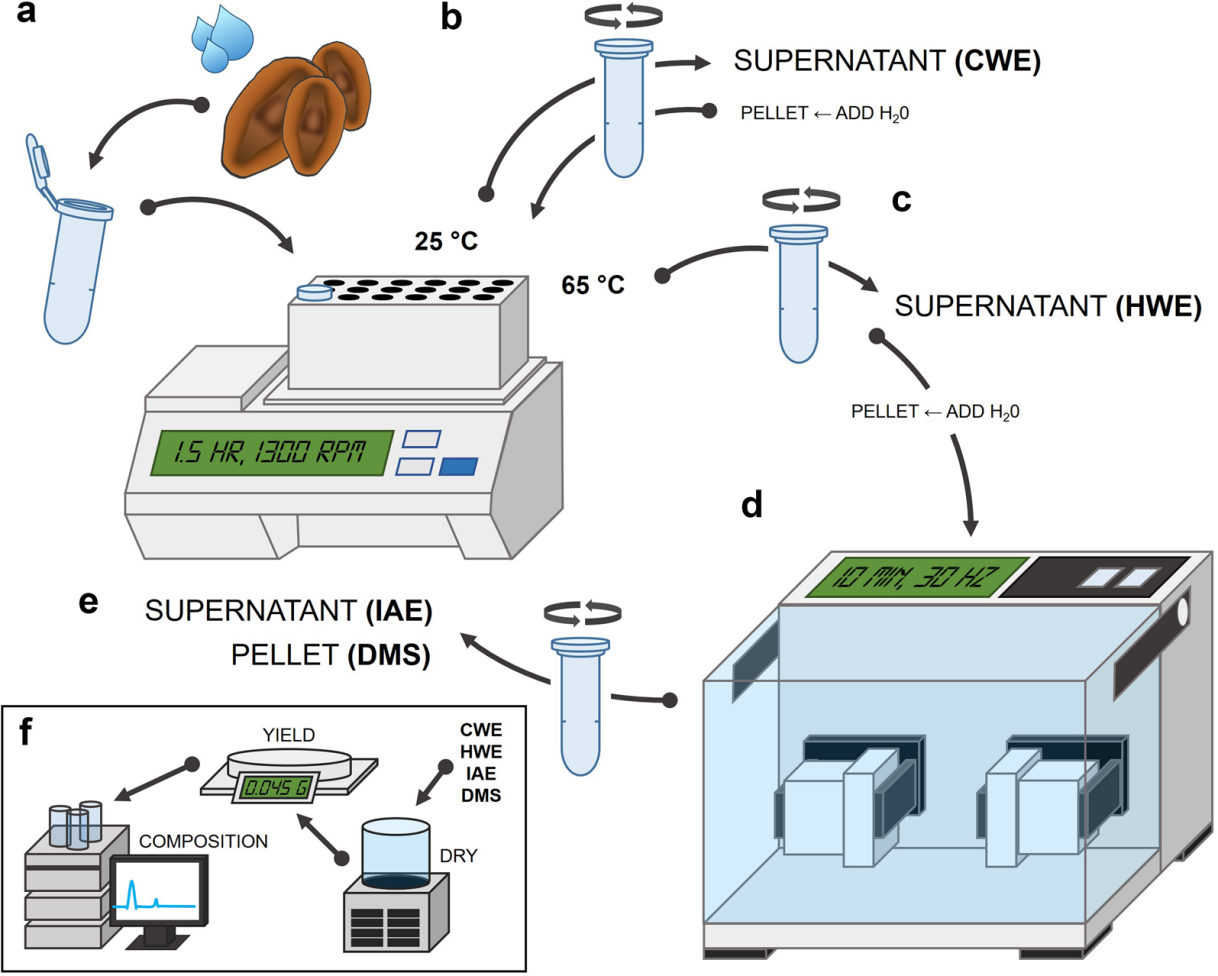
Schematic representation of pipeline used to extract small quantities of seed mucilage in discrete fractions for yield and chromatographic analysis. **a** Seeds and water are added to a microcentrifuge tube and incubated in a thermomixer-type shaking incubator at 25 °C for 1.5 h with agitation. **b** After centrifugation, the cold water extractable (CWE) mucilage is removed and water is added to the pellet to restore the working volume. **c** The sample is incubated at 65 °C for 1.5 h with agitation and after centrifugation the hot water extractable (HWE) mucilage is removed. Water is added to the pellet to restore the working volume. **d** Intense agitation by a mixer mill-type tissue disrupter is used to disrupt the seed-mucilage attachment. **e** After centrifugation, the intense extraction extractable (IAE) mucilage is removed. The pellet contains the demucilaged seeds (DMS). **f** All fractions can be freeze-dried for yield and chromatographic analysis

The rapid small scale fractionation of quantities of seed mucilage suitable for yield and chromatographic analyses was achieved using the following protocol incorporating elements of methods by Yu et al. [26] and Voiniciuc et al. [4]. For similarly sized seeds, seeds can be counted or for variably sized seeds 30 mg (± 0.5 mg) of seeds (exact mass recorded) can be weighed. Once the pre-extraction mass was recorded, seeds were added to a 2 mL microcentrifuge tube followed by 1.5 mL of RT DI H_2_O (Fig. 1a). Tubes were vortexed briefly to break surface tension and ensure all seeds are immersed. To obtain the first mucilage fraction (Fig. 1b), tubes were incubated for 1.5 h at 25 °C in a shaking incubator (Eppendorf ThermoMixer^®^ Comfort, Germany) with agitation at 1300 rpm then centrifuged (Eppendorf 5424, Germany) for 2 min at 13,000 rpm. Ensuring that the pelleted adherent mucilage and seeds are not disturbed, the tubes were removed from the centrifuge and using a 1000 μL laboratory pipette, the supernatant—the cold water extractable (CWE) mucilage fraction—was transferred to a clean, pre-labelled microcentrifuge tube. This transfer may require multiple steps based on the volume of the supernatant. The volume of the tube contents comprising the pelleted mucilage and seeds was returned to approximately 1.5 mL with RT DI H_2_O based on the tube markings (different samples may require slightly different volumes). To obtain the next mucilage fraction (Fig. 1c), a similar process was employed but at a warmer temperature: tubes were incubated for 1.5 h at 65 °C with agitation at 1300 rpm then centrifuged for 2 min at 13,000 rpm. Again the supernatant—the hot water extractable (HWE) mucilage fraction—was transferred to a clean, pre-labelled microcentrifuge tube using a 1000 μL laboratory pipette. After removal of the HWE fraction, the volume of the pellet is significantly reduced as only the most extraction-resistant mucilage remains tightly adhered to the seed. After adjusting the volume of tube contents to approximately 1.5 mL with RT DI H_2_O, the tubes were agitated intensely at 30 Hz for 10 min on a tissue disruptor-type mixer mill (Retsch MM400, Germany) using a microcentrifuge tube adapter (Fig. 1d). Tubes were centrifuged for 2 min at 13,000 rpm and the supernatant—the intense agitation extractable (IAE) mucilage fraction—was transferred to a clean, pre-labelled microcentrifuge tube using a 1000 μL laboratory pipette.

From each sample, a cold water extractable (CWE), hot water extractable (HWE) and intense extraction resistant (IAE) fraction of seed mucilage has been obtained along with the corresponding demucilaged seeds (DMS) (Fig. 1e). These four fractions were frozen at − 80 °C for 24 h and then freeze-dried (Labconco Freezone 6, US) to a constant weight. Freeze-dried mucilage and demucilaged seeds were transferred to a microbalance with 0.01 mg resolution (Shimadzu AUW220D, Japan) with fine-tip tweezers to calculate mucilage yield (Fig. 1f).

Yield of mucilage fractions can be calculated using the following equation:

$$ {\text{Yield}} \left( \% \right) = \left( {\frac{{{\text{mass\, of\,freeze\,dried\, mucilage}}}}{{{\text{mass\,of\,seeds\,pre-extraction}}}}} \right) \times 100. $$

*Optional*—isolated fractions may be pipetted into a 2000 μL 96 well deep well plate (Eppendorf, Germany) in place of new microcentrifuge tubes which can become unwieldy when dealing with large sample numbers. The deep well plates can accommodate many samples and several plates will fit simultaneously into a freeze-dried unit for bulk processing.

### Monosaccharide profiles of fractionated seed mucilage

Freeze-dried mucilage was dispersed in water at 2 mg/mL (w/v) and an 800 μL aliquot was added to 200 μL of 5 M H_2_SO_4_ (final H_2_SO_4_ concentration of 1 M) and hydrolysed at 100 °C for 3 h as per Phan et al. [30]. Monosaccharides released by acid hydrolysis were derivatised with 1-phenyl-3-methyl-5-pyrazoline (PMP) and then separated by reversed phase high performance liquid chromatography (RP-HPLC) following Comino et al. [31] with modifications to the column and eluents listed in Hassan et al. [32]. Area under the peaks was compared to standard curves of mannose, ribose, rhamnose, glucuronic acid, galacturonic acid, glucose, galactose, xylose, arabinose and fucose [33].

### Water absorption assay

After weighing 20 seeds into a 2 mL microcentrifuge tube, 1 g of water was added, and mucilage was allowed to expand undisturbed for 45 min at 25 °C. Using a 1 mL syringe without a needle, unabsorbed water was removed and weighed. Water absorption capacity can be calculated using the equation:

$$ {\text{Water}}\;{\text{absorption}}\;{\text{capacity}} \left( {\text{g/g}} \right) = \frac{{{\text{Initial}}\;{\text{weight}}\;{\text{of}}\;{\text{water}}\;{\text{added}} - {\text{weight}}\;{\text{of}}\;{\text{unabsorbed}}\;{\text{water}}}}{{{\text{Initial}}\;{\text{weight}}\;{\text{of}}\;{\text{seeds}}\;{\text{added}}}} $$

## Results and discussion

### This protocol achieves total mucilage extraction

**Fig. 2:**
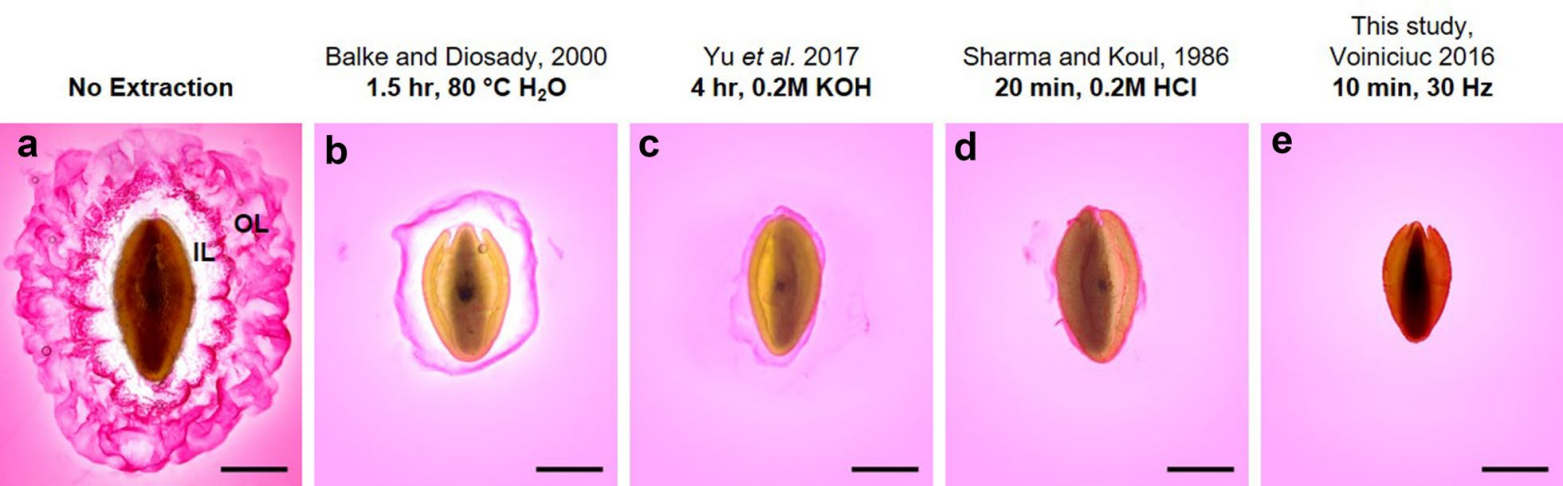
Seed mucilage that remains attached to *P. ovata* seeds after the application of various published extraction protocols and staining with 0.1% w/v ruthenium red. **a** The characteristic mucilage architecture of *P. ovata* seed mucilage. **b** After extended extraction in hot water the fine structure of the outer layer (OL) of mucilage has been lost leaving an inner adherent layer (IL). **c** The use of a chaotropic agent, KOH, significantly reduces the size of the mucilage envelope leaving only a film of mucilage that is resistant to chemical extraction. **e** Mucilage has been hydrolysed in situ and only a thin feathery layer remains. **e** Extraction by intense agitation leads to total mucilage removal. Scale = 1 mm

Figure 2 shows *P. ovata* seeds with stained expanded seed mucilage before and after four methods of mucilage extraction. While hot water was effective at reducing the size of the mucilage envelope (Fig. 2b), mucilage in the inner layer is more densely packed than the removed soluble fraction and thus a large amount of the mucilage remains [34]. Yu et al. [26] reported that an extended (4 h) extraction with 0.2 M KOH, a chaotropic agent, was sufficient to remove the adherent mucilage layer by disrupting hydrogen bonds in the mucilage. We confirm that this treatment is effective at removing the majority of seed mucilage (Fig. 2c) although the most strongly adherent portion remained on all treated seeds (n = 30). Mucilage removal in acid was similarly effective (Fig. 2d), however the mode of action of the acid was to hydrolyse the mucilage in situ which is not useful if any downstream functional or linkage analyses are required. An appealing alternative was the non-chemical extraction method devised by Voiniciuc et al. [4] who were able to efficiently extract all adherent mucilage from *Arabidopsis* seeds using intense agitation on a tissue disruptor-type mixer mill. The physical force of the shaking was sufficient to disrupt the mucilage-seed attachment and disperse the polysaccharides. We corroborate the efficacy of this method on *P. ovata* where close to 100% of seed mucilage was removed (Fig. 2e). We also observed that mucilage staining of seeds that sequentially underwent a hot water extraction before 0.2 M KOH, 0.2 M HCl or 30 Hz agitation were no different to those that were not extracted with hot water as a first step (data not shown).

The monosaccharide profiling of fractionated *P. ovata* mucilage shows that our small-scale fractionation technique is directly comparable to the larger scale technique published by Yu et al. [26], the original study by Guo et al. [35] on which their work is based and a similar work published earlier by Marlett and Fischer [36] (Additional file 1: Table S1).

### The extraction pipeline effectively provides material for identifying variation in seed mucilage characteristics

**Fig. 3:**
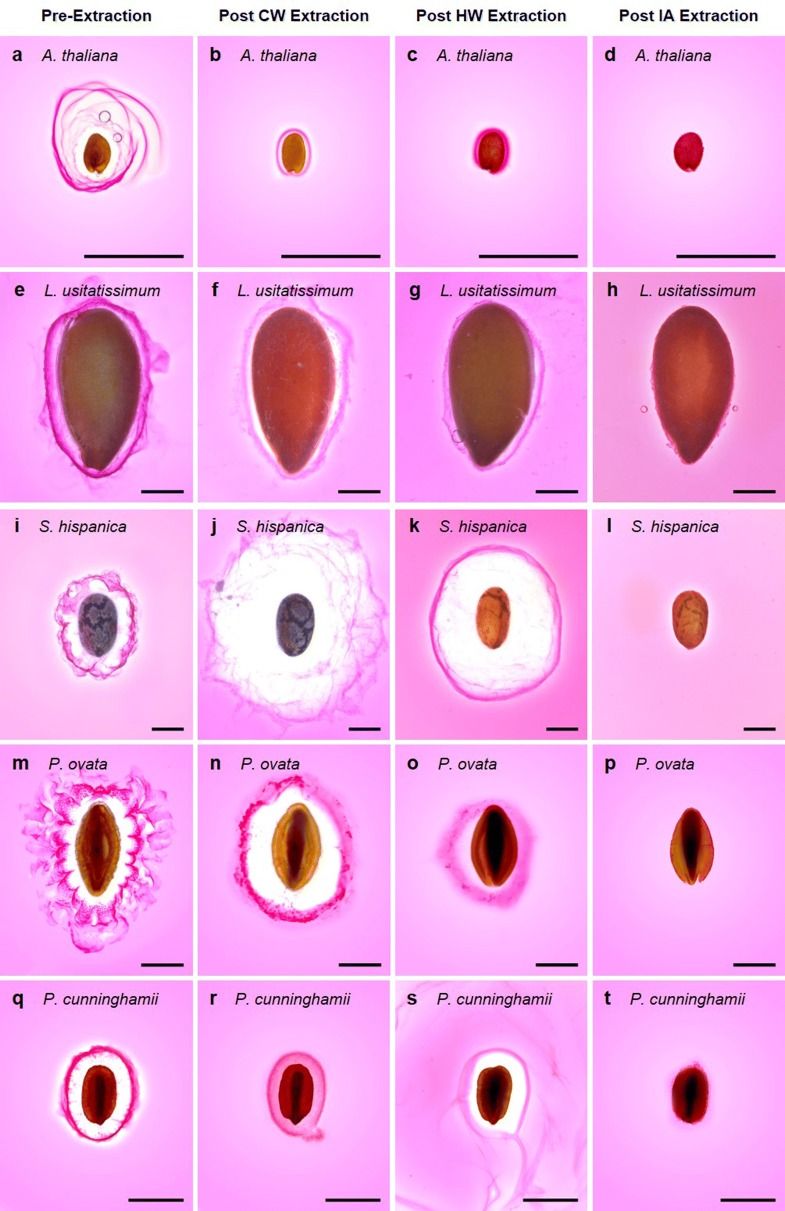
Visual inspection of the ruthenium red-stained seed mucilage of five myxospermous species (*Arabidopsis thaliana*,* Linum usitatissimum*, *Salvia hispanica*, *Plantago ovata* and *Plantago cunninghamii*) after sequential fractionation shows that the size of the mucilage envelope is sequentially changed corresponding to removal/dispersion of constituent polysaccharides. Note that images are not taken of the same seed as each seed was disposed of after imaging. Scale = 1 mm

**Fig. 4:**
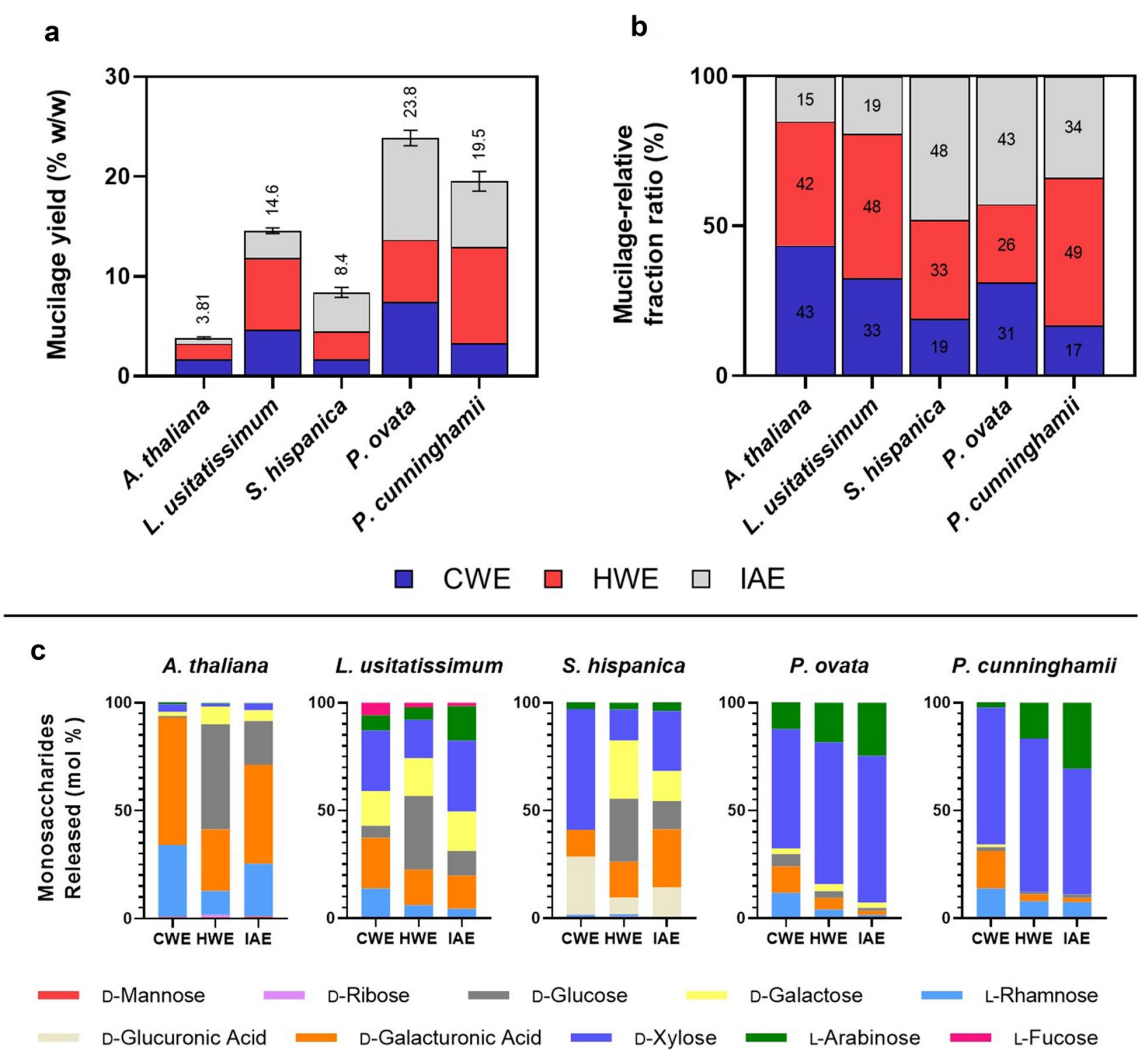
The extraction pipeline is effective at providing material suitable for identifying significant intergeneric and interspecific variation in seed mucilage yield, fractionation profile and composition. **a** Yield of total seed mucilage divided into constituent fractions. Error bars refer to standard deviation in total mucilage yield of five biological replicates. **b** Extractability of seed mucilage represented as the share of each fraction within total extracted mucilage. **c** Monosaccharide analysis of fractionated mucilage showing intergeneric, interspecific and intraspecific differences in mucilage chemical composition. Values presented are in molar ratio of quantified monosaccharides released by acid hydrolysis. *CWE* cold water extractable, *HWE* hot water extractable, *IAE* intense agitation extractable

Figure 3 shows the appearance of the expanded seed mucilage envelope of *A. thaliana* (a–d), *L. usitatissimum* (flaxseed) (e–h), *S. hispanica* (chia) (i–l), *P. ovata* (psyllium) (m–p) and an Australian native relative of psyllium, *P. cunninghamii* (q–t) before and after each mucilage fractionation step in the pipeline described here, which culminates in total extraction (Fig. 4a). After CWE and HWE extraction, the mucilage envelope of *L. usitatissimum* and *A. thaliana* is significantly reduced in size compared to *S. hispanica*, *P. ovata* and *P. cunninghamii*. These changes were reflected in the differences between the ratios of extracted fractions (Fig. 4b), where *L. usitatissimum* and *A. thaliana* were most susceptible to extraction, yielding the largest proportion of water extractable (CWE + HWE) components. The easy removal of the delicate outer layer of mucilage in *A. thaliana* and *L. usitatissimum* is consistent with previous findings and established fractionation techniques [4, 34, 37–40]. Contrastingly, *Plantago* and *S. hispanica* mucilage has been reported to require more effort to efficiently extract total mucilage. For *S. hispanica*, mucilage extraction in cold water is not efficient [41] while extended hot water extractions yield only slightly greater quantities [42–44]. We corroborate these findings, reporting that only half of the mucilage is extractable by hot water (Fig. 4b). Efficient mucilage extraction from *Plantago* species is also difficult, often requiring multiple physical and/or chemical extraction steps [26, 35, 36, 45, 46]. While total yields of mucilage between *P. ovata* and *P. cunninghamii* were similar (Fig. 4a), there are clear interspecific differences in the relative proportion of each fraction (Fig. 4b). Changes in appearance of the mucilage envelope of *P. ovata* after CWE were more noticeable than for *P. cunninghamii*, which appears relatively unchanged and is reflected in CWE yield which was lower for *P. cunninghamii.* Yield of HWE mucilage was greater for *P. cunninghamii*, with less IAE mucilage than *P. ovata.*

Some, if not all, of the differences in the relative proportion of each mucilage fraction of the species studied can be ascribed to mucilage polysaccharide composition and the associated difference in properties. Monosaccharide analysis confirmed significant differences in mucilage composition between genera and species and their isolated mucilage fractions (Fig. 4c). Monosaccharide analysis also confirmed previous findings that mucilage fractions from *A. thaliana* and *L. usitatissimum* are rich in rhamnose and galacturonic acid [4, 47–50], components of pectin, a highly water-soluble polysaccharide, the presence of which may contribute to overall ease of extraction in these species. It is the presence of minor mucilage components in the HWE and IAE fractions that are known to affect the mucilage properties including fractionality. In *A. thaliana*, monosaccharide profiling of the HWE and IAE fractions (containing the adherent mucilage) confirms previous findings of a molar reduction in rhamnose and galacturonic acid residues and an increase in non-cellulosic glucose, mannose, galactose and xylose [49, 50], components of minor polysaccharides like xylan and glucomannan that are well-known to interact with and tether the adherent mucilage at the seed surface [4, 7, 51–53]. Similarly, rhamnose and galacturonic acid residues were reduced in the HWE and IAE fractions of *L. usitatissimum*, along with increases in xylose and arabinose residues associated with heteroxylan, known to significantly alter the functional properties of RG-I [47]. In *P. ovata and P. cunninghamii*, the three mucilage fractions contained high levels of xylose and arabinose (heteroxylan) with a smaller amount of rhamnose and galacturonic acid (pectin), congruent with previous findings by Phan et al*.* [30]. Like both *A. thaliana* and *L. usitatissimum*, pectin-associated monosaccharides are enriched in the CWE fractionation and diminish with further fractions. While the presence of pectin has been proposed to modulate the extractability of the major heteroxylan component in *Plantago* mucilage, studies have shown that heteroxylan branching has the most significant influence on the mucilage properties including the extractability [26–28]. In both *P. ovata* and *P. cunninghamii,* the ratio of arabinose to xylose residues (estimation of the degree of sidechain branching) increased with resistance to extraction, in line with those studies. However, more explicit structural characterisation will be needed to define the fine structure and its relationship to interspecific differences in extractability. The mucilage of *S. hispanica* is unique among the species studied in that its constituent polysaccharide(s) have not been found in any other genera [54]. Its unique structure containing xylose, glucose and glucuronic acid residues is consistent with the monosaccharide data (Fig. 4c), although the molar ratios between the constituents varies by fraction suggesting that fine structure and/or interactions with minor components (from which the other monosaccharides detected are derived) influences the extractability.

### The pipeline has utility in quality testing of mucilaginous species

**Fig. 5:**
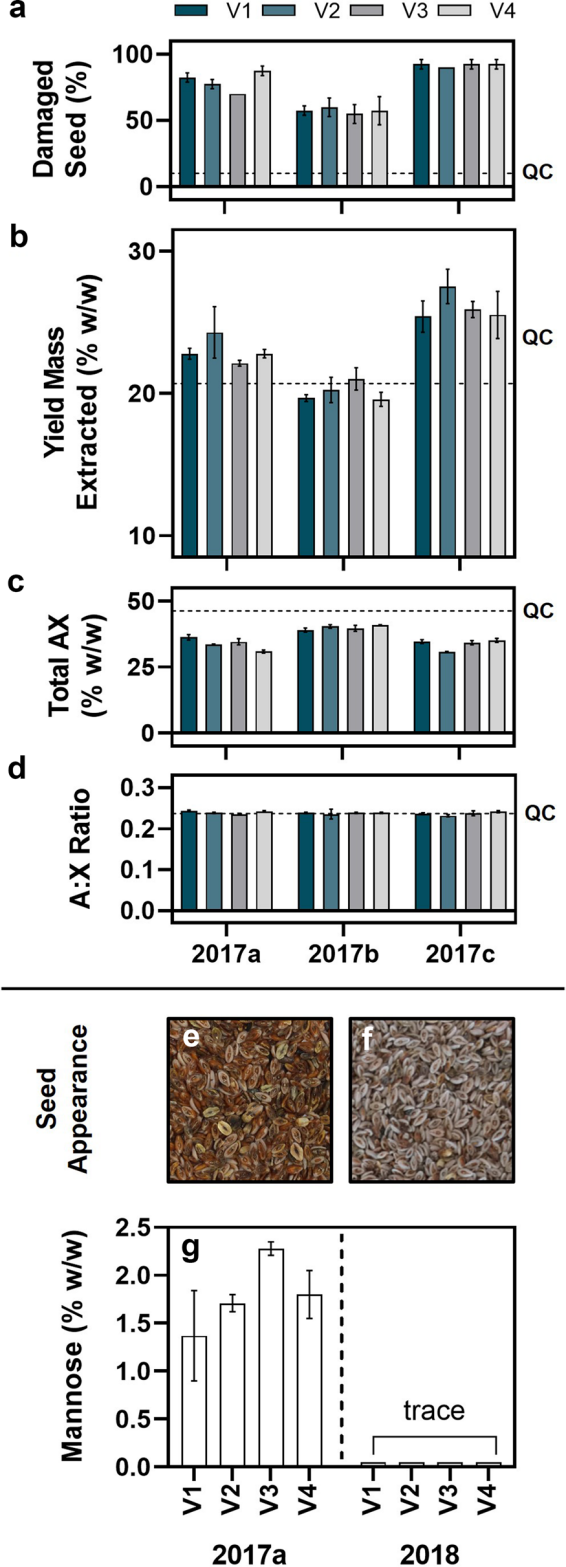
In field-grown samples of *Plantago ovata*, the extraction pipeline has utility in quality testing when coupled with yield and chromatographic analysis. **a** Visual inspection of damaged seed provides a baseline quality score. **b** Yield of total mucilage varied most significantly between times of sowing with a minor intervarietal effect. **c** Heteroxylan content (the proportion of arabinose and xylose residues in extracted mucilage) inversely related to mucilage yield and seed quality. **d** The ratio of arabinose to xylose residues in extracted mucilage (an approximation of heteroxylan branching) remains unchanged indicating that seed and mucilage development were unperturbed. **e** Seed grown at trial sites in 2017 is unevenly coloured, with many blackened seeds while, **f** seeds grown at the same site in 2018 are more consistent, with the light-coloured husk material consistently visible on seeds. **g** In the 2017 field trials (analysed are samples from 2017a), significant quantities of mannose were identified by monosaccharide analysis in extracted mucilage of each variety grown indicative of seed damage-related endosperm leakage. Contrastingly, only trace amounts (well below the limit of quantitation) were found in the same varieties grown in the following year, 2018. Dotted line in **a**–**d** indicates the value of a quality control sample (QC). Error bars represent one standard deviation (some are small and not easily visible)

The production of high-quality psyllium gum from *P. ovata* seeds is hampered by agronomic issues which cause poor quality, damaged seeds [55]. Damaged seed coat allows leakage of endosperm components during extraction which alter the functional properties and cause significant discolouration due to phenolic browning which is undesirable in many applications (Cowley et al*.* unpublished data). As the functional component of psyllium gum, heteroxylan must be abundant and present at a consistent level to be considered good quality for industrial uses as the dilution of heteroxylan by the presence of contaminants will impact the functionality in optimised formulations. Four varieties of *P. ovata* were grown in three separate field trials with different times of sowing a factor which, due to climatic conditions, was found to significantly affect seed quality [55, 56]. A suite of quality parameters is shown in Fig. 5. Visual inspection was used to determine a baseline quality score for the four varieties at each trial (Fig. 5a). Trial 2017a had the lowest damage score followed by 2017b and then 2017c. 2017c was deemed the poorest quality with consistently high seed damage. When mucilage was extracted using our pipeline, there were clear differences in yield, with the strongest effect related to time of sowing with only minor intervarietal influence (Fig. 5b). Varieties grown at 2017b did not differ greatly in yield from the control, which was a high-quality field grown sample (QC). Conversely, varieties from 2017a and 2017c had higher mass yields after extraction. Monosaccharide profiling showed that mucilage synthesis was not disrupted as the arabinose to xylose (AX) ratio was very similar between varieties and trials and not significantly different from the QC (Fig. 5d). However, the quantity of heteroxylan in the mucilage (defined as total AX) differed between trials (Fig. 5c). 2017b, confirmed as the most successful sample, had the highest proportion of AX and was closest to the QC. Correspondingly, 2017a and 2017c had lower proportions of AX indicating significant contamination from other components. Total AX is thereby inversely proportional to yield as a direct result of quality. No variety at any trial had AX as high as the QC, likely because seed of the QC sample was of exceptional quality.

Furthermore, known chemical markers have been defined indicating low quality or damaged seed. As one example, extreme damage of *P. ovata* seeds causes extensive leakage of endosperm components including mannose monosaccharides (Cowley et al. unpublished data). Trial 2017a was impacted by devastating unseasonable rainfall which physically damaged seed before harvesting (Fig. 5e) and subsequently led to microbial growth, leading to detectable quantities of mannose in the extracted mucilage (Fig. 5g). Mannose was found only in trace amounts in corresponding 2018 samples which were not weather damaged or microbially contaminated and more consistently high quality (Fig. 5f).

### The pipeline can be used for rapid screening of myxospermous germplasm

**Fig. 6:**
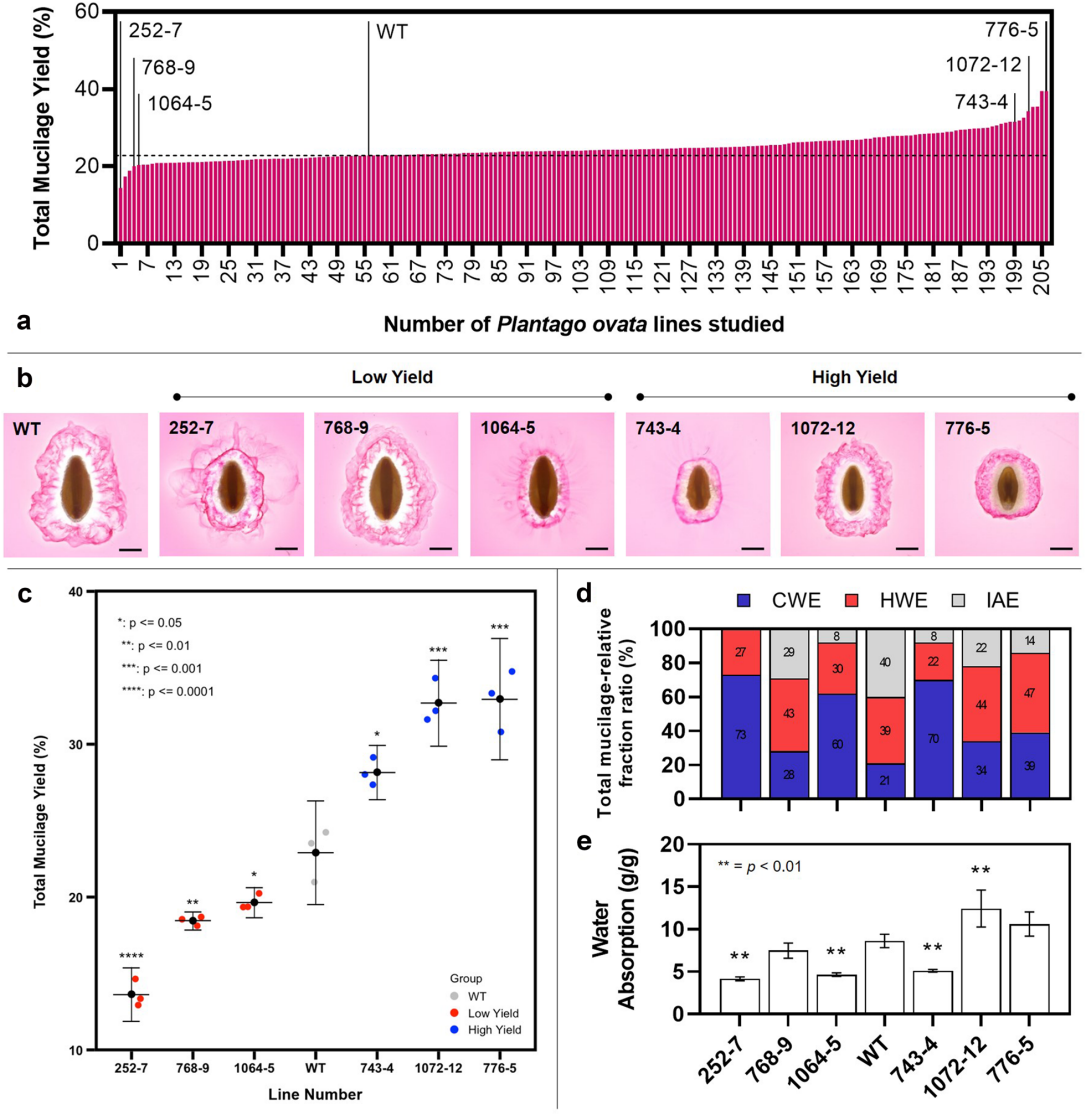
The extraction pipeline described here has utility in screening myxospermous germplasm. **a** Total seed mucilage yield of 206 *Plantago ovata* mutants screened using the extraction pipeline. **b** Mucilage architecture of a validation set including a wild type, three low yield and three high yield lines observed using ruthenium red staining (scale = 1 mm). **c** Verification of mucilage yield in the validation set. **d** Differences in the seed mucilage fractionation profile of the validation set. **e** Water absorption by seed mucilage of the validation set. Values in **c** and **e** are means while error bars represent one standard deviation from the mean. Asterisks denote statistical differences from WT (significance levels are included on the relevant figures) as determined by Student’s t-test

This pipeline has utility for rapid screening of mucilage yield traits in a germplasm set, demonstrated here using gamma-irradiated *P. ovata* mutants generated in a previous study [10]. Total mucilage yield data (a pooling of CWE, HWE and IAE fractions) was obtained for a subset of 206 randomly-selected glasshouse-grown *P. ovata* mutants (Fig. 6a). In 63% of mutants, mucilage yield was within a ± 10% interval of WT yield (n = 131). Only 4% of mutants yielded 10% less mucilage than WT (n = 8), while 33% yielded over 10% more (n = 68). To validate this screen, a subset of the three lowest yielding (252-7, 768-9, and 1064-5) and three highest (743-4, 1072-12, and 776-5) mutants were selected for further analysis. Expanded mucilage architecture has been used previously to visually screen for altered mucilage phenotypes in mutants of *Arabidopsis* [4, 57, 58] and *Plantago* [10]. Here we show variation in expanded mucilage architecture between the mutants (Fig. 6b) where some are distinctly different to WT (252-7, 1064-5, 743-4, and 776-5) while others are WT-like (768-9 and 1072-12). The utility of the pipeline is proven two-fold in that it can identify highly-distinctive mutants which would be identified through typical visual screening techniques (like ruthenium red staining) but also mutants with more subtle changes to yield that may appear as WT. The validation set of mutant lines was subjected to further analysis which confirmed the differences observed in total mucilage yield were statistically significant compared to the WT. Differences observed in the size of the ruthenium red-stained mucilage envelopes and the amount of mucilage extracted may be linked to alterations in polysaccharide macromolecular properties. This was examined further by comparing the relative proportion of the three mucilage fractions (Fig. 6d) with the water absorption capacity of the mucilage (Fig. 6e).

While the total yield of mucilage was significantly decreased from WT in mutant 768-9, the ruthenium red phenotype, the ratio of mucilage fractions and the water absorption capacity were not significantly different from the WT. Contrastingly, the ratio of the three mucilage fractions was significantly altered in mutants 252-7, 1064-5, and 743-4 (*mucilage extractable with water* (*mew*) mutants) where the CWE and HWE fractions comprise most or all of the mucilage and the IAE fraction is significantly diminished or totally absent. In *mew* mutants, water absorption capacity is significantly reduced from the WT presumably due to a reduction in the stronger gelling, high water-holding capacity IAE mucilage fractions [26]. The striking similarities in the phenotypes of the *mew* mutants suggests that they may contain mutant alleles. Importantly, *mew* mutant 252-7 has already been identified as a putative reduced mucilage xylan mutant [10] and the characterisation of this class of mutant is ongoing [11]. Mutant 776-5 represents a previously unseen class of *P. ovata* mucilage mutant [10, 11, 59]. While this mutant has the highest total mucilage yield in the screened population, its water absorption capacity was unchanged from WT. Its unique compact ruthenium red phenotype and shift in the ratio of the three mucilage fractions suggests intrinsically different changes to the mucilage composition, with a novel causative mutation(s) compared to the *mew* mutants. The ease of distinguishing the *mew* mutants and mutant 776-5 within the mutant population shows that the pipeline can effectively identify putative mutants with perturbed seed development and/or mucilage synthesis, ideal for forward genetic studies.

In contrast to the *mew* mutants and mutant 776-5, it was found that while the ruthenium red and mucilage fractionation phenotype of mutant 1072-12 did not differ substantially from the WT, the total yield and related water absorption capacity was significantly increased. Mutant 1071-12 may therefore represent an important genotype for use in pre-breeding efforts due to its high mucilage yield without the aberrant changes to mucilage composition and properties which makes other mutants less suitable.

## Conclusions

In this study we tested the efficacy of several established protocols for seed mucilage extraction and downsized and adapted the most effective elements into a small-scale, rapid extraction and analysis pipeline. We demonstrated the utility of this pipeline for investigating intergeneric and interspecific differences in seed mucilage characteristics, as well as for quality testing and germplasm screening of myxospermous plants. This pipeline is already regularly used in our research group increasing the analysis efficiency of a range of myxospermous species. It has also been adopted by a leading food manufacturer who relies on consistently high-quality mucilage products. The use of this pipeline in fundamental research may improve our understanding of mucilage production and properties, ensure quality in food manufacturing, and aid in pre-breeding or breeding of myxospermous species—often classified as orphan crops—that could benefit from improved characterisation methods.

## Supplementary information

**Additional file 1: Table S1. **Monosaccharide summary of *Plantago ovata *mucilage fractionated by the small-scale extraction pipeline in comparison with previously published large scale techniques.

## Data Availability

The datasets used and analysed during this work are available from the corresponding author upon reasonable request.

## Abbreviations

CWE: Cold water extractable
HWE: Hot water extractable
IAE: Intense agitation extractable
DI water: Deionized water
RG-I: Rhamnogalacturonan I
QC: Quality control
A:x Ratio: Arabinose to xylose ratio
AX: Arabinose + xylose (estimated heteroxylan content)
WT: Wild type
