# Supplementary material for: A small-scale fractionation pipeline for rapid analysis of seed mucilage characteristics
Source: Plant Methods. 2020 Feb 24;16:20. doi: 10.1186/s13007-020-00569-6 (PMC7038624; doi:10.1186/s13007-020-00569-6)
Supplement: Supplementary file 1 — Additional file 1: Table S1. Monosaccharide summary of Plantago ovata mucilage fractionated by the small-scale extraction pipeline in comparison with previously published large scale techniques. [file 13007_2020_569_MOESM1_ESM.pdf]

Additional File 1: Table S1. Monosaccharide summary of *Plantago ovata* mucilage fractionated by the small-scale extraction pipeline in comparison with previously published large scale techniques.

<table>
<tr><th>Fraction</th><th>Marlett & Fischer 2002[a]</th><th>Guo et al. 2008[b]</th><th>Yu et al. 2017[c]</th><th>This study</th></tr>
<tr><td colspan="5">Heteroxylan-Relative Pectin Content (Rha+GalA:Xyl Ratio)</td></tr>
<tr><td>CWE</td><td>0.679</td><td rowspan="2">0.286</td><td>0.426</td><td>0.438</td></tr>
<tr><td>HWE</td><td>trace R+G</td><td>trace R+G</td><td>trace R+G</td></tr>
<tr><td>IAE</td><td>-</td><td>trace R+G</td><td>trace R+G</td><td>trace R+G</td></tr>
<tr><td colspan="5">Heteroxylan Branching (Ara:Xyl Ratio)</td></tr>
<tr><td>CWE</td><td>0.200</td><td rowspan="2">0.232</td><td>0.211</td><td>0.222</td></tr>
<tr><td>HWE</td><td>0.285</td><td>0.301</td><td>0.281</td></tr>
<tr><td>IAE</td><td>-</td><td>0.345</td><td>0.341</td><td>0.364</td></tr>
</table>

a. From the work of Marlett and Fischer, psyllium husk fractions Fr C and Fr B are deemed comparable to CWE and HWE fractions, respectively. Their remaining fraction, Fr A, contained alkali insoluble husk material and thus is not directly comparable to the IAE fraction.
b. From Guo *et al.*'s work, psyllium husk fraction WE is deemed to be similar to a pooling of CWE and HWE while AEG 0.5 is deemed comparable to IAE
c. From Yu *et al.*'s work CW, HW and KOH fractions are deemed comparable to CWE, HWE and IAE fractions, respectively.
